# Peri-implantitis progression and modulating factors: evidence synthesis and implications for clinical management

**DOI:** 10.1590/1807-3107bor-2026.vol40suppl1065

**Published:** 2026-08-21

**Authors:** Cátia Sufia Alves Freire de ANDRADE, João Pedro dos Santos SILVA, Gildenilson OLIVEIRA, Bruna Egumi NAGAY, Jamil Awad SHIBLI, João Gabriel Silva SOUZA, Valentim Adelino Ricardo BARÃO

**Affiliations:** (a) Universidade Estadual de Campinas - Unicamp, Piracicaba Dental School, Department of Prosthodontics and Periodontology, Piracicaba, SP, Brazil; (b) Universidade de São Paulo - USP, Bauru School of Dentistry, Department of Prosthodontics and Periodontology, Bauru, SP, Brazil; (c) Faculdade Israelita de Ciências da Saúde Albert Einstein, Hospital Israelita Albert Einstein, São Paulo, Brazil; (d) Universidade de Guarulhos, Dental Research Division, Guarulhos, SP, Brazil

**Keywords:** Peri-Implantitis, Dental Implants, Risk Factors, Biofilms

## Abstract

Peri-implantitis is a biofilm-related disease characterized by inflammation and tissue damage in the peri-implant mucosa, along with progressive loss of supporting bone. Although triggered by a polymicrobial biofilm, disease progression is influenced by factors beyond microbial buildup. This thorough review explores factors that may promote peri-implant disease progression, focusing on interactions among biofilm dynamics, local conditions, patient-related factors, and material-related characteristics. Evidence suggests that biofilm maturation, ecological imbalance, and ongoing inflammatory response are key drivers of disease progression. Locally, microbial shifts, iatrogenic conditions, and reduced cleanability seem to facilitate microbial buildup and hinder maintenance. Patient factors such as a history of periodontitis, smoking, and poorly controlled diabetes are most consistently linked to poorer peri-implant outcomes, while genetic susceptibility remains suggested but not confirmed. Material properties, including surface topography, roughness, chemistry, wettability, corrosion behavior, and particle release, may influence microbial colonization and host responses, though their independent clinical significance is less certain. Overall, current research points to a multifactorial model of peri-implant disease progression, though much of the evidence is based on observational studies with substantial heterogeneity, limiting the ability to draw definitive conclusions. A comprehensive understanding of microbial, biological, prosthetic, systemic, and material factors is crucial for accurate risk assessment, prevention, and personalized management of peri-implantitis.

## Introduction

The use of osseointegrated dental implants has become a well-established approach for oral rehabilitation, offering predictable functional and esthetic outcomes in partially and completely edentulous patients.^1^ Despite the high clinical success rates of implant therapy, biologic complications remain a major challenge, particularly peri-implant diseases, which can compromise peri-implant tissue stability and long-term treatment outcomes.^2^ Among these conditions, peri-implantitis is currently recognized as a biofilm-induced pathological condition characterized by inflammation of the peri-implant mucosa, accompanied by progressive loss of supporting bone, which is considered the main reason for treatment failure.^3^ Since the 2017 World Workshop, this definition has provided the conceptual basis for diagnosis, epidemiologic interpretation, and clinical decision-making in implant dentistry.^3^

Although peri-implantitis is initiated by polymicrobial biofilm, its progression cannot be explained by microbial accumulation alone. Polymicrobial biofilm accumulation on implant devices acts as a chemical and physical "stress factor" to the surrounding tissues, triggering host inflammatory responses that lead to disease onset and tissue damage.^4^ As observed on tooth surfaces, continuous biofilm accumulation is associated with a microbial shift toward a more pathogenic profile and a pro-inflammatory environment, resulting in sustained disease progression.^5^ As stated in the latest consensus on peri-implant diseases, peri-implantitis is always preceded by peri-implant mucositis, a condition in which inflammation is confined to the peri-implant mucosal tissues.^3^ Therefore, if not controlled or treated, the disease progresses to peri-implantitis. Both host-related and microbial factors influence the severity of peri-implant tissue breakdown. Notably, histological evidence indicates that peri-implantitis lesions exhibit more extensive tissue destruction compared with periodontitis.^6^

Biofilm accumulation and, in particular, its compositional shifts are directly modulated by various factors, including extracellular matrix content, sugar exposure, the inflammatory microenvironment, and products released from implants, among others.^7^ The disease is now understood as a dynamic and multifactorial process in which polymicrobial biofilm maturation, ecological imbalance, host inflammatory responses, and environmental changes interact over time to sustain tissue breakdown^8^. In this context, the transition from peri-implant health to mucositis and, eventually, to peri-implantitis appears to be modulated by a wide range of local, systemic, and material-related factors that may influence biofilm retention, tissue stability, host susceptibility, and maintenance efficacy.

Over the last few years, several factors have been investigated as possible modulators of peri-implant disease progression. However, interpreting these factors remains challenging.^8^,^9^ Much of the available evidence is derived from observational studies, frequently affected by heterogeneity in case definitions, differences in patient populations, incomplete control of confounding variables, and variations in prosthetic and maintenance protocols.^10^ Moreover, in terms of mechanisms and modulating factors, *in vitro* and *in vivo* evidence has also provided important insights into disease progression at the microbiological and molecular levels. Consequently, while some factors are consistently associated with worse peri-implant outcomes, the strength and significance of these associations are not always consistent, and the distinction between true causal determinants and context-dependent risk indicators is often unclear.^9^,^10^ Therefore, this critical review discusses the main factors involved in the onset and progression of peri-implant disease, emphasizing the interplay among polymicrobial biofilm, local predisposing conditions, patient-related factors, and material- or prosthesis-related influences. It aims to provide a clearer understanding of why peri-implantitis develops and progresses differently across clinical scenarios, and why its management requires attention not only to microbial control, but also to the broader biologic and restorative context in which implant therapy is delivered.

For this, a critical narrative review was conducted, including a literature search in PubMed/MEDLINE, Scopus, and Web of Science, along with complementary manual screening of reference lists from relevant publications. The search strategy combined controlled terms and free-text keywords related to peri-implantitis and its modulating factors ("peri-implantitis", "peri-implant disease", "disease progression", "biofilm", "risk factors", "local factors", "patient-related factors", "implant surface", and "material-related factors"), using Boolean operators (AND, OR) to combine the main concepts across databases. Priority was given to systematic reviews, meta-analyses, consensus reports, clinical studies, and translational evidence relevant to the biological and clinical interpretation of peri-implant disease progression. The evidence was analyzed narratively, considering study relevance, biological plausibility, and consistency across studies.

## Main mechanisms and modulating factors favoring disease progression

**Figure 1:**
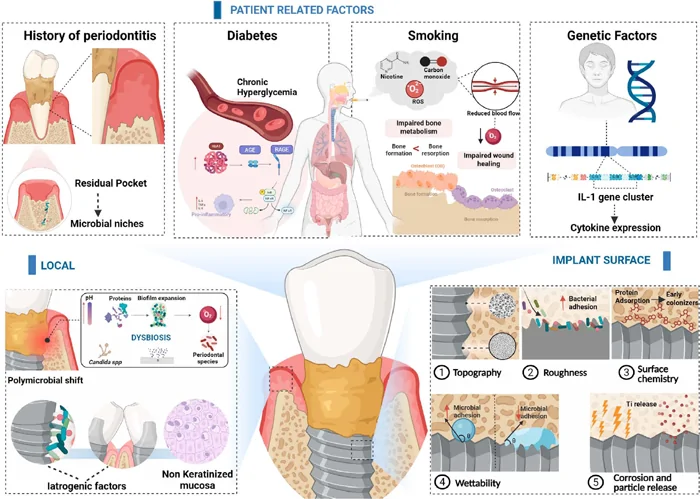
Schematic overview of the main factors associated with peri-implant disease progression. Patient-related factors, including a history of periodontitis, diabetes, smoking, and genetic susceptibility, may increase host susceptibility and impair tissue homeostasis. Local factors, such as polymicrobial shift, excess cement, iatrogenic conditions, and non-keratinized mucosa, may favor plaque retention and compromise peri-implant tissue stability. Implant surface characteristics, including topography, roughness, surface chemistry, wettability, and corrosion/particle release, may further modulate microbial adhesion and host responses. Created with BioRender.com (license number: LV29LBP3MX).

Peri-implant disease progression should be interpreted as the result of a complex and bidirectional interaction between biofilm-induced inflammation and a range of modulating factors that may influence tissue breakdown over time. While the accumulation and maturation of polymicrobial biofilm represent the central biological basis for triggering the inflammatory process and initiating disease, available evidence also suggests that local microbial-retentive conditions, patient-related factors, and implant physicochemical characteristics may influence disease onset, persistence, severity, and possible progression (Figure 1). In this context, peri-implant tissue destruction arises not from a single determinant but from the cumulative, often interrelated effects of factors that alter microbial ecology, impair host-implant homeostasis, and compromise peri-implant health. Thus, a comprehensive understanding of disease progression depends on examining how these mechanisms converge and reinforce one another in clinically relevant scenarios. The factors are supported by varying degrees of evidence, ranging from consistent clinical associations to findings based mainly on biological plausibility or limited data. Factors such as a history of periodontitis, smoking, poor glycemic control, biofilm-retentive local conditions, and impaired cleansability are more consistently supported by clinical evidence as clinically relevant modifiers of peri-implant outcomes. In contrast, genetic susceptibility, peri-implant keratinized mucosa as an independent determinant, and specific implant surface-related characteristics are supported more variably and should be interpreted more cautiously, often considering biological plausibility, translational evidence, or limited and heterogeneous clinical findings.

### Polymicrobial biofilms as key drivers of peri-implant disease

**Figure 2:**
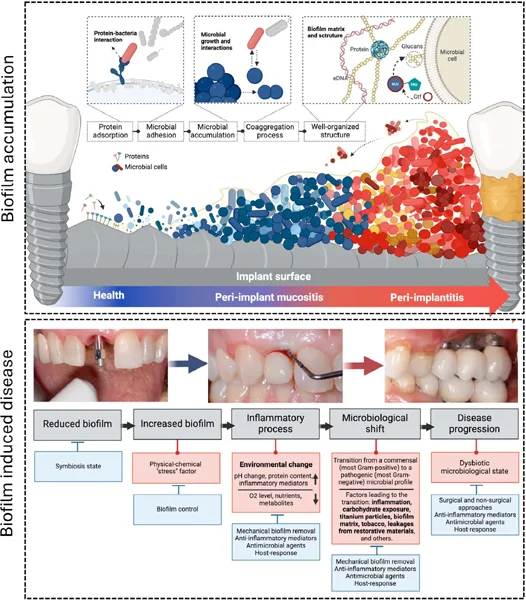
Schematic representation of polymicrobial biofilm accumulation and biofilm-induced peri-implant disease progression. Protein adsorption onto the implant surface promotes early microbial adhesion, followed by microbial accumulation, coaggregation, and biofilm maturation. As the biofilm becomes more structured and resistant, inadequate biofilm control favors inflammation, ecological changes, dysbiosis, and progression from peri-implant health to peri-implant mucositis and peri-implantitis. This schematic representation was reprinted and adapted under the terms of the Creative Commons CC BY license (open access).^7^

A polymicrobial biofilm is the main biological driver of peri-implantitis (Figure 2).^3^ The process begins with the rapid adsorption of salivary and crevicular proteins onto the implant surface, forming a conditioning layer that facilitates early microbial adhesion.^11^ Importantly, surface physicochemical properties modulate the protein adsorption profile, with implant surfaces differing from enamel and dentine.^12^ Once this pellicle is established, pioneer microorganisms adhere to the surface and interact with one another, leading to microbial accumulation, coaggregation, and progressive biofilm maturation.^13^ Although peri-implantitis and periodontitis present similar microbial profiles in terms of composition, we recently showed that titanium (an implant-based material) differs from enamel and dentine in terms of initial bacterial attachment and biofilm formation, including co-aggregation processes and early microbial shifts, highlighting the role of surface properties in modulating polymicrobial biofilm accumulation and progression.^12^,^14^ As the biofilm develops, microorganisms become embedded in an extracellular matrix composed of extracellular polysaccharides and other polymeric components, which stabilizes the three-dimensional structure and increases resistance to mechanical removal, host defenses, and antimicrobial agents.^15^,^16^

Under health-associated conditions, this biofilm may remain compatible with a symbiotic host-microbiome relationship.^17^ However, when biofilm control is insufficient, biomass increases and the peri-implant environment is exposed to persistent physicochemical and biological stress, triggering mucosal inflammation and the onset of peri-implant mucositis.^3^ In turn, inflammation alters local ecological conditions and favors the selection of a more pathogenic microbial community.^18^ Changes in nutrient availability, inflammatory mediators, and the local peri-implant environment promote a shift from health-associated communities to a dysbiotic microbiota enriched in gram-negative anaerobes and other disease-associated taxa.^19^ This transition may be further intensified by sucrose exposure, cross-kingdom microbial interactions, titanium particle and ion release, and other factors.^15^,^16^ Therefore, peri-implantitis should not be interpreted as a simple consequence of microbial accumulation, but rather as a dynamic process in which biofilm maturation, environmental changes, dysbiosis, and host responses progressively reinforce one another and sustain tissue destruction.^3^,^18^ Thus, local factors, aspects related to patients' health conditions, and implant-based material properties have been suggested to modulate the etiologic factor—polymicrobial biofilms—and disease progression and will be further detailed in this review (Figure 1).

### Local factors

**Figure 3:**
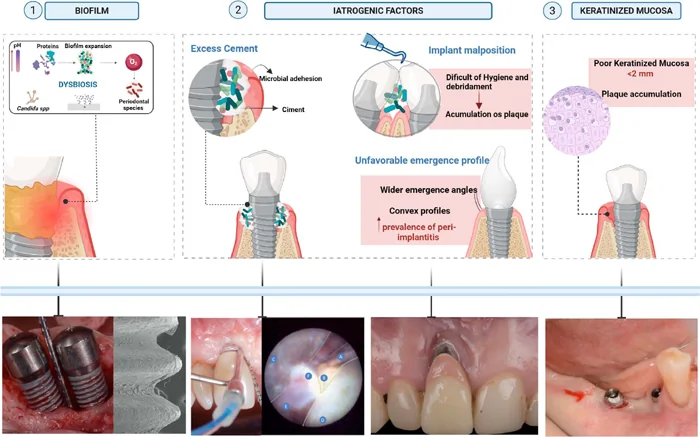
Schematic representation of the main local factors associated with the onset and progression of peri-implant diseases.

Clinical signs of implant-related diseases and their progression directly reflect increasing bio film accumulation on implants.^5^ Thus, local factors at the implant site or in the oral environment are expected to directly affect microbial accumulation and composition, often leading to disease progression through their effects on host inflammatory responses (Figure 3). Among local factors, polymicrobial shifts and iatrogenic conditions that impair cleansability appear to be more consistently supported as clinically relevant modifiers of peri-implant outcomes, whereas the role of reduced keratinized mucosa seems more dependent on its effects on brushing comfort, plaque control, and tissue stability than on a clearly established independent effect on peri-implantitis.

(1) Biofilm and polymicrobial shift: inflammatory changes in the peri-implant environment increase pH and nutrient availability, promoting biofilm expansion, reduced oxygen levels, and the overgrowth of anaerobic and pathogenic species, leading to dysbiosis. (2) Iatrogenic factors: residual cement acts as a plaque-retentive surface, facilitating microbial adhesion and biofilm accumulation; implant malposition and unfavorable prosthetic design, including wider emergence angles and convex profiles, impair oral hygiene and debridement, favoring plaque accumulation and increasing the risk of peri-implantitis. (3) Keratinized mucosa: reduced width of keratinized mucosa (<2 mm) is associated with plaque accumulation and compromised peri-implant tissue maintenance. Representative clinical images illustrating each condition are shown in the lower panel. The schematic illustrations were created with BioRender.com (License number: JS29LBPGQJ). Panels 1^29^, 2^45^, 3^28^, and 4^46^ were reprinted and adapted under the terms of the Creative Commons CC BY license (open access).

#### Polymicrobial shift

Biofilm biomass and shifts in its microbial composition are directly influenced by several factors, mainly related to changes in the oral environment and at the implant site. Inflammatory processes in the surrounding peri-implant tissues alter the local microenvironment, increasing pH, protein, and nutrient availability, thereby promoting the overgrowth of proteolytic and putative pathogens highly associated with tissue damage.^20^,^21^ Although inflammation has been recognized as a key factor leading to microbiological shifts in implant-related biofilms, as also observed in periodontitis, other factors related to biofilm composition and the oral environment may also drive the system toward a dysbiotic state. Extracellular polymers synthesized by bacterial exoenzymes can increase biofilm biomass and alter biofilm structure, thereby reducing oxygen levels and, consequently, favoring the growth of anaerobic and putative pathogens, such as red complex periodontal species.^15^ These polymers are primarily synthesized from sucrose, and sucrose exposure has been shown to induce microbiological shifts in biofilms on titanium surfaces.^22^

Although bacterial content has been attributed to a key role in triggering inflammatory responses and inducing disease, cross-kingdom interactions with *Candida spp.* may also contribute to disease progression.^23^ Although *Candida* may not act as an etiological factor, its presence may promote bacterial growth, increase extracellular polymer content, and contribute to tissue damage. However, its role in the progression and modulation of peri-implantitis has not been widely explored in the literature. In addition to microbiological factors, products released from implant materials, such as ions and particles, have been suggested to chemically and physically promote biofilm biomass and contribute to the overgrowth of putative pathogens.^24^,^25^ However, most of this evidence derives from *in vitro* and *in situ* studies. It remains unclear whether peri-implantitis affects the overall oral microbiome, including the salivary microbiome.^18^ Nevertheless, changes in the oral microbiome may favor the accumulation of bacterial species on implant surfaces, including periodontal pathogens. The presence of other biofilm-induced diseases in the oral cavity or at sites with greater microbial accumulation may also serve as reservoirs that elevate pathogen levels in the oral environment, thereby promoting colonization of dental implants and potentially modulating disease progression.^26^,^27^

#### Iatrogenic factors

Among local iatrogenic factors, peri-implant disease progression may also be favored by excess cement, implant malposition, deep submucosal restorative margins, overcontoured prostheses, unfavorable emergence profiles or angles, and implant-abutment or transmucosal component choices that reduce tissue stability or limit maintenance access.^28^,^29^

Excess cement has been consistently discussed as a potential local risk indicator for peri-implant diseases, with several observational studies reporting an association between residual cement and peri-implant mucositis or peri-implantitis.^30^,^31^ Even so, the frequency of diseased sites in which cement remnants are detected varies markedly across studies, and not all implants with excess cement develop disease.^30^ In parallel, comparative studies have not consistently shown a greater risk of peri-implantitis in cement-retained restorations than in screw-retained restorations, suggesting that the retention mode itself is not the sole determinant of biologic complications.^32^ A plausible explanation for the observed association is that residual cement may act as a rough, biofilm-retentive surface, favoring microbial colonization and maturation.^31^ In addition, the likelihood of undetected cement increases as restorative margins are positioned deeper beneath the mucosa, making removal more difficult.^33^ Some authors have also suggested that cement composition may influence tissue response, with zinc-containing cements potentially exhibiting greater solubility and a less adverse tissue response than other materials.^34^,^35^ Overall, the available evidence supports the interpretation that excess cement should be regarded as a potential risk indicator for peri-implantitis, although the relationship remains mainly observational rather than definitive causal evidence.^30^,^31^,^32^

When the implant is placed in a prosthetically unfavorable position, the definitive restoration often requires a bulky or overcontoured transmucosal shape to re-establish the correct crown position, which may impair patient-performed oral hygiene and hinder professional instrumentation during maintenance.^28^,^29^ Likewise, deep restorative margins and non-cleansable contours may create protected areas for biofilm stagnation, making early inflammatory changes more difficult to detect and manage clinically.^28^,^29^ Current evidence therefore suggests that the biologic relevance of these iatrogenic factors lies mainly in their effects on cleansability, biofilm control, and peri-implant soft-tissue stability rather than in any single technical feature.^28^,^29^ Clinical evidence has shown that inadequate access to peri-implant hygiene is associated with increased bleeding on probing and a higher plaque index.^36^ In this context, emergence profile and emergence angle have received particular attention, because observational studies and systematic reviews indicate that convex profiles and wider emergence angles may be associated with a higher prevalence of peri-implantitis, although the strength of this evidence remains limited and context-dependent.^37^,^38^,^39^ This caution is important because the clinical significance of a wider emergence angle likely depends on the overall restorative design, implant platform configuration, and the extent to which the final contour interferes with hygiene access.^37^,^39^ In addition, implant-abutment and transmucosal component selection may influence peri-implant tissue behavior, since connection design and component configuration can affect mucosal integration and early marginal bone stability.^40^ Overall, iatrogenic factors should be interpreted as treatment-related conditions that become clinically relevant when they create a prosthetic environment that favors persistent biofilm accumulation and complicates maintenance over time.^28^,^29^

#### Keratinized mucosa

Current evidence suggests that peri-implant keratinized mucosa is primarily relevant for biofilm control, soft-tissue stability, and maintenance, rather than as a consistently proven independent determinant of peri-implantitis. Sites with a keratinized mucosal width <2 mm may cause brushing discomfort, which can impair oral hygiene and favor biofilm accumulation, thereby compromising peri-implant tissue maintenance.^41^,^42^ An umbrella review found that <2 mm of keratinized mucosa was associated with increased biofilm accumulation, gingival inflammation, mucosal recession, and marginal bone loss, but not clearly with bleeding on probing, probing depth, implant survival, or disease prevalence.^42^ Accordingly, the most balanced interpretation is that adequate keratinized mucosa appears to improve cleansability and peri-implant tissue stability, whereas its independent role in determining peri-implantitis remains less definitive.^43^ In line with this, current EFP guidance states that the keratinized/attached mucosal width should be recorded after prosthesis delivery, and that augmentation may be considered in cases of absent keratinized/attached mucosa and discomfort during brushing.^44^

### Patient-related factors

#### History of periodontitis

In implant dentistry, a history of periodontitis has been consistently associated with an increased risk of peri-implantitis.^47^ Evidence from umbrella reviews and consensus reports supports periodontitis as one of the most relevant patient-related factors associated with peri-implant outcomes. This association should be interpreted considering differences in exposure definitions across studies, since some reports refer to a documented history of periodontitis, whereas others reflect persistent periodontal instability over time. From a clinical perspective, the most relevant interpretation is that periodontitis, particularly when associated with residual inflammation or residual periodontal pockets, may increase susceptibility to peri-implant tissue breakdown.^48^,^49^ In parallel, both the 2025 AO/AAP consensus and the 2017 World Workshop on the Classification of Periodontal and Peri-Implant Diseases and Conditions recognized a history of periodontitis as a major risk factor for peri-implant diseases. 8,47

This association appears to be mediated less by the previous diagnosis itself than by persistent periodontal instability over time, since residual periodontal pockets after active therapy may function as persistent inflammatory and microbial niches and have been associated with peri-implantitis, implant loss, and worse peri-implant parameters.^50^,^51^ Biologically, these residual pockets may harbor dysbiotic biofilms and sustain chronic inflammatory activation, thereby increasing susceptibility to peri-implant tissue breakdown over time. However, the reported risk magnitude remains variable across studies, and differences in case definitions, disease severity, and maintenance-related variables likely contribute to this heterogeneity. The effect of a history of periodontitis appears to be strongly modulated by residual inflammation and maintenance compliance rather than by the previous diagnosis alone. Moreover, periodontitis directly affects the oral microbiome, which may influence the accumulation and overgrowth of pathogenic species on implant surfaces.

#### Diabetes

Some evidence supports an association between diabetes/ hyperglycemia and peri-implantitis, although this association is not equally consistent across all peri-implant conditions or study designs.^47^ The 2025 AO/AAP consensus on prevention and management of peri-implant diseases and conditions identified uncontrolled diabetes as a high-risk factor/indicator for peri-implantitis progression, and recent evidence syntheses suggest that the association is more consistent with peri-implantitis than with peri-implant mucositis.^47^ This pattern also appears to be influenced by metabolic control, since worse peri-implant parameters and greater marginal bone loss have been more frequently reported in patients with higher HbA1c levels.^52^ However, the association between diabetes and peri-implantitis is still based on limited evidence and contradictory results. While some systematic reviews indicate diabetes as a risk factor for peri-implantitis and worse clinical signs, others suggest that the evidence for this association is still inconclusive and requires further evaluation.^48^,^53^

Biologically, chronic hyperglycemia may impair wound healing, delay osseointegration, alter bone turnover, and intensify inflammatory dysregulation through pathways involving advanced glycation end products (AGEs) and their receptors. In line with this, diabetes/hyperglycemia has been associated with increased levels of AGEs, IL-6, TNF-α, IL-8, and RANKL in peri-implant crevicular fluid, while probing depth and marginal bone loss tend to worsen as HbA1c increases.^54^,^55^ Despite this biological plausibility, the available clinical evidence still relies predominantly on observational studies, often with heterogeneous case definitions, limited metabolic characterization, and incomplete adjustment for relevant confounders, including smoking, history of periodontitis, and adherence to maintenance therapy. For this reason, diabetes is better interpreted as a modifier of peri-implant risk rather than as an isolated or uniformly acting determinant.

#### Smoking

Smoking is a modifiable behavioral factor that has long been investigated in relation to peri-implant diseases.^8^ Earlier evidence regarding its association with peri-implantitis risk was often considered inconclusive due to heterogeneity in study design, case definitions, and control of confounding variables.^8^ More recent evidence syntheses, however, have strengthened this association. In this context, the 2025 AO/AAP consensus identified smoking as a major systemic and behavioral risk factor for peri-implant diseases,^47^ but this association should not be interpreted simplistically, since the tobacco-related effect appears to vary according to current smoking status and cumulative exposure.

Biologically, smoking may impair peri-implant health through vascular, immunologic, microbiological, and bone-related effects. Cigarette smoke contains substances such as nicotine and carbon monoxide, which exert local vasoconstriction, impair leukocyte function, and reduce blood oxygenation, while also negatively affecting bone metabolism and the bone-implant interface.^56^ From a microbiological perspective, smokers tend to harbor more anaerobic pathogens and have lower microbial diversity, which may favor the transition from peri-implant health to disease.^48^ Thus, smoking is better interpreted as a modifiable factor that may increase susceptibility to peri-implant breakdown and disease progression, rather than as an isolated or uniformly acting determinant.

#### Genetics

Peri-implantitis has been investigated in relation to genetic susceptibility, since genetic polymorphisms may influence host inflammatory and bone-related responses.^48^ Historically, most studies have focused on inflammatory markers, and the association of genetics with peri-implantitis is particularly linked to polymorphism activity, such as those in the IL-1 gene cluster; other candidates, such as OPG, CD14, TNFα, and IL-6, have also been explored.^8^ These polymorphisms may influence susceptibility to peri-implantitis by modulating cytokine expression, inflammatory signaling, and bone-related pathways, thereby favoring a dysregulated host response, extracellular matrix breakdown, osteoclast activity, and peri-implant bone loss.^57^ More recently, it was shown that the most frequently investigated markers involve inflammatory cytokines, bone metabolism, and immune regulation, with more consistent signals for IL-1β +3954 C/T, CD14 rs2569190, and MMP-8 variants, whereas findings for TNF-α -308 G/A remained inconsistent across populations.^57^ However, this body of evidence remains far from definitive, as no quantitative metaanalysis could be performed and substantial heterogeneity persisted across genetic targets, ethnicity, diagnostic criteria, and control of confounding factors. Therefore, current genetic evidence is more consistent with host susceptibility than with an established clinical risk factor for disease progression. Among patient-related factors, a history of periodontitis, smoking, and poor glycemic control are more consistently supported by clinical evidence as relevant modifiers of peri-implant outcomes, whereas genetic susceptibility remains a suggestive but less clinically established factor.

### Material-related factors

Dental implant surface properties are increasingly recognized as important factors influencing interactions among the biomaterial, oral microbiota, and peri-implant tissues. While systemic and local patient-related factors remain key determinants of successful rehabilitation, the physicochemical features of implant surfaces also affect biological responses, ranging from osseointegration to peri-implant disease. In this context, growing attention has been given to surface characteristics such as morphology, topography, roughness, wettability, and electrochemical stability, since these properties can impact initial bacterial adhesion and subsequent biofilm development. Although most commercially available implant surfaces were originally designed to improve osseointegration, current evidence indicates they may also affect microbial colonization. Therefore, implant surface properties might influence the balance between peri-implant homeostasis and dysbiosis, potentially affecting the development and progression of peri-implant diseases.

Overall, these material-related characteristics should be interpreted mainly as biologically plausible modulators of microbial colonization and host responses, whereas their independent clinical relevance to peri-implant disease progression remains less definitive than that of patient-related and cleansability-related factors. The following sections discuss the main surface-related characteristics associated with this process.

#### Surface morphology, topography, and roughness

The surface architecture of dental implants comprises three closely related physical characteristics: morphology, topography, and roughness.^58^ Together, these features define the geometric organization of the implant surface at the interface with surrounding tissues. Historically, early dental implants had relatively smooth, low-roughness machined surfaces, reflecting the technological limitations of the time. The pioneering studies by Brånemark in the 1960s established the biological basis of osseointegration by demonstrating direct integration between titanium implants and bone tissue.^59^ In later decades, experimental and clinical evidence showed that surface modifications could enhance implant-bone interactions, leading to the development of new surface treatment strategies.^60^ As a result, most commercially available implants now present moderately rough surfaces (Sa > 1.0- ≤ 2.0 um) with distinct morphological patterns. Surface treatments generate microtextured surfaces and increase the effective area available for bone contact. Subsequently, manufacturers have also introduced hydrophilic surfaces to improve early biological interactions.^60^

Although these modifications were originally designed to improve implant stability and osseointegration, increasing evidence indicates that surface architecture may also affect microbial colonization.^61^ Topographical irregularities enlarge the contact area for physicochemical interactions and may favor bacterial retention by creating protected niches that hinder biofilm removal. In this context, rougher surfaces may facilitate the initial adhesion of pioneer microorganisms and promote peri-implant biofilm maturation, a process closely linked to the development and progression of peri-implantitis.^61^,^62^ Surface topography may also influence the intermolecular forces involved in bacterial adhesion, since micro- and nanoscale structures modify contact conditions and affect van der Waals, hydrophobic, and electrostatic interactions.^58^ Once established, this biofilm may sustain persistent inflammation in peri-implant tissues.

Advances in biomaterials engineering have enabled the development of more complex surfaces that combine micro- and nanoscale features to simultaneously modulate cellular and microbiological responses. Plasma Electrolytic Oxidation (PEO), for example, has been explored to produce highly porous and bioactive surfaces with interconnected micropores.^63^ Recent evidence also suggests that PEO-treated surfaces may exert sustained antimicrobial effects over time, potentially reducing the risk of peri-implant disease development.^64^ However, surface architecture is not static. Mechanical wear, corrosion, and clinical manipulation may alter roughness and topography over time, affecting surface physicochemical properties and, consequently, biological interactions at the peri-implant interface.^65^ These changes may favor bacterial retention or alter the local inflammatory response, thereby contributing to disease progression.

Overall, surface morphology, topography, and roughness should be understood as integrated components of the functional architecture of implants. By simultaneously influencing cellular adhesion, bone formation, and microbial colonization, these properties may affect the balance between osseointegration and microbial dysbiosis. Thus, although micro- and nanostructured surfaces were developed to improve implant bioactivity, their topographical features may also contribute to biofilm persistence and peri-implant inflammation, both of which are directly involved in the etiopathogenesis and progression of peri-implantitis.^64^ However, evidence on how physical properties mediate microbial adhesion and accumulation indicates that this interaction is influenced not only by surface properties but also by microbial cell characteristics, with specific effects varying by bacterial species.^7^ Moreover, it remains unclear whether a cause-and-effect relationship exists between certain properties, such as surface roughness, and increased biofilm progression or microbiological shifts, since, after the initial adhesion phase, other factors modulate microbial composition and growth.

#### Surface chemistry, free energy, and wettability

Surface chemistry and surface free energy play a central role in peri-implant biology because they influence the initial adsorption of proteins, wettability, and interactions between the implant surface and blood and host cells. Thus, surface chemistry should not be viewed as merely a passive property of the material, but as an active modulator of the implant-tissue-cell interface. On titanium implants, the spontaneously formed TiO2 layer provides biocompatibility and relative chemical stability; however, even small changes caused by industrial treatments, surface aging, atmospheric contamination, or clinical handling may alter surface free energy and wettability patterns.^60^ For this reason, different commercial surface treatments have been developed to modulate these properties and optimize early tissue response. More hydrophilic surfaces are often associated with improved blood wetting, protein adsorption, and early cellular activity, which may support faster osseointegration.^58^ However, within the oral cavity, the same surface is rapidly covered by an acquired pellicle, which may mask part of the intrinsic properties of the material, such as surface free energy.

This issue becomes particularly relevant in peri-implantitis, since a higher free-energy surface may favor host responses but may also modify protein adsorption patterns and, indirectly, influence the adhesion of early microbial colonizers. Therefore, the key clinical question is not simply whether a surface is hydrophilic or hydrophobic, but whether its chemistry helps maintain a microenvironment that favors tissue sealing while limiting the persistence of dysbiotic biofilms over time. This is especially important in the transmucosal region, where the quality of soft tissue sealing may influence bacterial penetration and the chronicity of peri-implant inflammation.^3^

From a clinical perspective, however, the currently available evidence does not support the notion that a specific commercial surface, solely on the basis of its chemistry or wettability, consistently reduces the incidence or severity of peri-implantitis.^2^ In fact, a clinical study comparing OsseoSpeed and SLActive implants found no significant association between the implant system and the occurrence or severity of peri-implantitis. In contrast, factors such as plaque control and male sex appeared more relevant.^66^ Overall, surface chemistry and wettability should be interpreted as modulating rather than deterministic factors in peri-implant disease progression.

Although the ideal surface would combine high initial bioactivity, low pathogenic pellicle retention, chemical stability over time, resistance to organic contamination, and effective support for osseointegration, this biological optimum has not yet been clearly defined. Moreover, this limitation becomes even more relevant because implant surfaces must also remain electrochemically stable in the oral environment, where pH fluctuations, biofilm accumulation, cyclic loading, and implant-abutment interactions may disrupt the passive oxide layer and favor corrosion, tribocorrosion, and the release of ions or metallic particles into peri-implant tissues.

#### Electrochemical behavior and particle release

Although titanium is highly corrosion-resistant due to its passive titanium dioxide (TiO2) layer, this protection may be compromised under oral conditions, including pH fluctuations, bacterial biofilms, changes in saliva composition, cyclic loading, and micromovements at the implant-prosthetic interface. Together, these factors may promote corrosion and tribocorrosion, in which chemical degradation and mechanical wear occur simultaneously, leading to disruption of the oxide layer and the release of metallic ions or titanium particles.^67^,^68^ These processes may affect both the implant surface and mechanically stressed areas, such as the implant-abutment interface.

Titanium particles and ions have been identified in peri-implant tissues, suggesting that degradation products can accumulate in the local inflammatory environment. 65 Experimental studies indicate that these particles may be phagocytosed by macrophages, stimulating the release of pro-inflammatory mediators such as TNF-α, IL-1β, and IL-6, which may enhance RANKL expression, osteoclast differentiation, and bone resorption.^3^ In addition, corrosion-related changes in surface chemistry may alter surface energy and reactivity, favoring bacterial adhesion and the persistence of dysbiotic biofilms. Despite this evidence, the exact role of corrosion and the release of metallic particles in the etiopathogenesis of peri-implantitis remains under investigation. However, previous evidence showed that titanium particles and ions, as well as their combination, increase biofilm biomass and promote a dysbiotic state, including the overgrowth of red periodontal complex species.^24^ Although the precise roles of corrosion and particle release in peri-implantitis remain under investigation, current evidence suggests that these phenomena may act as modulators, intensifying inflammation and chemically promoting biofilm accumulation. Therefore, implant surface properties should be understood as interacting dynamically with microbial and host-related factors, influencing the balance between osseointegration and peri-implant disease progression.

## Limitations and future directions

This review has some limitations that should be acknowledged. As a critical narrative review, it does not follow a fully systematic methodology, which may increase the risk of selection bias and limit reproducibility. In addition, a substantial part of the available evidence on peri-implant disease progression is based on observational studies, particularly cross-sectional and retrospective designs, which restrict causal inference and often reflect disease prevalence rather than true longitudinal progression. Accordingly, some of the factors discussed in this review should be interpreted more appropriately as risk indicators or modulators associated with disease presence or severity, rather than as definitive determinants of longitudinal progression. Moreover, most evidence focusing on these mechanisms comes from *in vitro, in situ,* and animal models. The interpretation of the literature is further complicated by heterogeneity in case definitions, implant systems, prosthetic protocols, maintenance regimens, and adjustment for confounding variables. Moreover, several potentially relevant factors are highly interdependent, making it difficult to isolate the independent contribution of each variable to peri-implant breakdown. Therefore, the interpretations proposed in this review should be regarded as biologically plausible and clinically informative, but not as definitive proof of causality.

Future research should prioritize well-designed prospective cohorts and interventional studies that use standardized case definitions, a clearer distinction between disease onset and progression, and more consistent control of major confounders, such as smoking, diabetes control, history of periodontitis, and adherence to maintenance therapy. Greater effort is also needed to quantify local and prosthetic exposures, including restorative margin depth, emergence profile, cleansability, residual cement, and peri-implant keratinized mucosa, so that their clinical effects can be compared more reliably across studies. In parallel, translational research should further investigate how implant surface chemistry, topography, corrosion behavior, and particle release interact with dysbiotic biofilms and host immune responses over time. Future evidence syntheses would also benefit from stratifying findings by study design and level of evidence, thereby helping to distinguish true causal determinants from context-dependent risk indicators. Importantly, evidence regarding tooth-related biofilm and periodontitis has advanced over several decades, whereas concern about peri-implantitis is more recent. Therefore, knowledge derived from dental surfaces cannot be simply translated to dental implants without experimental validation. Since substrate surface properties mediate even the initial steps of biofilm formation, including protein pellicle composition and bacterial attachment^12^, it is expected that although peri-implantitis and periodontitis share similarities, disease progression and modulating factors may differ. Such knowledge is essential for developing strategies to prevent and control peri-implantitis and improve its management, particularly given that it is a prevalent condition that still lacks effective therapeutic approaches. Future evidence should also elucidate how these factors influence subsequent tissue healing following disease treatment, as well as their impact on the outcomes of bone tissue engineering approaches aimed at restoring peri-implant health and stability.^69^

From a clinical perspective, the multifactorial nature of peri-implant disease supports an individualized and multidimensional approach to risk assessment rather than reliance on isolated determinants. In this context, structured frameworks may help integrate relevant parameters, including history of periodontitis, bleeding on probing, residual pockets/probing depths, and prosthesis-related factors, into a comprehensive clinical risk profile. For this purpose, the Implant Disease Risk Assessment (IDRA)^70^ tool integrates eight clinical, patient-related, and prosthesis-related risk factors to estimate the likelihood of developing peri-implantitis, classifying patients as low, moderate, or high risk. This clinical tool is available online and can be accessed by clinicians to support risk estimation in daily practice. Although several of these factors are not yet supported by robust clinical or longitudinal evidence, the current, albeit limited, evidence suggests their association with peri-implantitis occurrence. Moreover, such tools require continuous updating as new evidence emerges, and future models should incorporate additional determinants, including material-related properties and systemic conditions. This approach may facilitate the identification of modifiable risk factors and improve risk communication during implant therapy and maintenance. However, individualized, patient-centered strategies remain essential, given the multifactorial nature of disease onset, progression, and severity.

## Conclusion

In conclusion, peri-implant disease progression cannot be explained by biofilm accumulation alone. Although polymicrobial biofilm remains the central biological driver, disease progression appears to result from interactions among dysbiosis, host inflammatory susceptibility, local prosthetic and iatrogenic conditions, and material-related characteristics that influence tissue stability and maintenance access. The current literature more consistently supports the role of factors such as history of periodontitis, smoking, diabetes control, biofilm-retentive local conditions, factors that modulate biofilm accumulation and composition, and inadequate cleansability, as clinically relevant modifiers of peri-implant outcomes, whereas the independent effects of other variables, including keratinized mucosa, genetics, and specific surface properties, remain less definitive. Importantly, much of the available evidence is observational, laboratory-based, and heterogeneous, limiting causal interpretation and reinforcing the need for cautious clinical extrapolation. Therefore, prevention and management of peri-implant disease progression should not rely on a single-factor model but rather on an individualized, multidimensional approach that integrates biofilm control, maintenance adherence, prosthetic planning, systemic risk assessment, and careful consideration of implant-related features.

## Data Availability

The authors declare that all data generated or analyzed during this study are included in this published article.
